# Supplementary material for: Realization of acoustic spin transport in metasurface waveguides
Source: Nat Commun. 2020 Sep 18;11:4716. doi: 10.1038/s41467-020-18599-y (PMC7501247; doi:10.1038/s41467-020-18599-y)
Supplement: Supplementary file 1 — Supplementary Information [file 41467_2020_18599_MOESM1_ESM.pdf]

Supplementary Information -
Realization of Acoustic Spin Transport in Metasurface Waveguides

Long et al.

CONTENTS

Supplementary Note 1. Improvements of spin values near boundaries 2

Supplementary Note 2. Selective excitations of metasurface waveguide modes. 2

Supplementary Note 3. SAM precession and additional phase 2

Supplementary Note 4. Robustness of transport protected by the SAM 3

Supplementary Note 1. IMPROVEMENTS OF SPIN VALUES NEAR BOUNDARIES

In this section, we would like to clarify the details that why our analytical theory gives imperfect spin values near the boundary in Fig. 3 of the main text. The reason is that our analytical theory in the main text is based on the lowest order trunction $l = 0, \pm 1$. If we incorporate more higher orders, namely $l = 0, \pm 1, \pm 2, ..., \pm \ell_{\max}$, the pressure field will be generally represented as: $p = p_0 \sum_{l=-\ell_{\max}}^{\ell_{\max}} c_l J_l(\kappa r) e^{il\varphi} e^{i(kz-\omega t)}$. One can then obtain the coefficients c_l according to boundary conditions in Fig. 1 of the main text. For the cases considering more higher order $\ell_{\max}$, we illustrate the theoretical pressure fields $|p|^2$ and spin angular momentum densities s_y in Supplementary Figure 1. Compared with simulation results, we can see that the analytical theory with the higher order $\ell_{\max}$ will give more accurate results than the lower orders $l = 0, \pm 1$. The SAM values near the boundary will be gradually close to simulation results. However, considering lowest orders is sufficient to express the SAM values in a neat analytical form, which clearly reflects our main points: non-zero SAM and tight spin-momentum locking.

Supplementary Note 2. SELECTIVE EXCITATIONS OF METASURFACE WAVEGUIDE MODES.

In this section, we will discuss the selective excitations of metasurface waveguide modes. As mentioned in Fig.1 of main text, the metasurface waveguide modes will have the spin-related selective excitations in Supplementary Figure 2(a): different directions for opposite spin excitations. However, due to dispersive resonant structures, the waveguide modes will not be perfect circularly polarized velocity fields. As shown in Supplementary Figure 2(b), for modes with $k_z > 0$ in $[2.85, 2.95]$kHz, it is clear that the velocity field at the center of the waveguide will have anticlockwise elliptically polarized forms, corresponding to the positive spin angular momentum density $s_y > 0$. These generally elliptically polarized velocity fields will make influences to the excitation efficiency due to the imperfect mismatch with spin sources (circularly polarized dipoles).

These momentum-related elliptically polarized velocity fields will give insights about the physical mechanism behind low scattering behaviours in Fig. 5 of main text. The modes of different directions will have opposite spin textures or opposite elliptically polarized fields, which results in low couplings (overlaps) between forward and reflected wave modes. These features would make back-scatterings suppressed. However, it is worth noting that the coupling between the opposite modes is small (but non-zero) due to their not-perfect-orthogonal elliptical polarizations, and therefore backscattering is weak but non-zero.

Supplementary Note 3. SAM PRECESSION AND ADDITIONAL PHASE

The variation of acoustic SAM texture in xy plane will follow the rotating evolution of velocity polarized profile as ($\frac{\partial}{\partial z}\boldsymbol{e}_i = \boldsymbol{\Omega}\times\boldsymbol{e}_i$): $\frac{\partial}{\partial z}\boldsymbol{s} = \frac{\rho_0}{2\omega}\frac{\partial}{\partial z}\mathrm{Im}[\boldsymbol{v}^*\times\boldsymbol{v}] = \frac{\rho_0}{2\omega}\mathrm{Im}[\frac{\partial \boldsymbol{v}^*}{\partial z}\times\boldsymbol{v}+\boldsymbol{v}^*\times\frac{\partial \boldsymbol{v}}{\partial z}] = \frac{\rho_0}{2\omega}\mathrm{Im}[(\boldsymbol{\Omega}\times\boldsymbol{v}^*)\times\boldsymbol{v}+\boldsymbol{v}^*\times(\boldsymbol{\Omega}\times\boldsymbol{v})] = \frac{\rho_0}{2\omega}\mathrm{Im}[\boldsymbol{\Omega}\times(\boldsymbol{v}^*\times\boldsymbol{v})] = \boldsymbol{\Omega}\times\boldsymbol{s}$. For the total SAM, $\boldsymbol{S} = \int \boldsymbol{s}d\boldsymbol{r}^2$, we can obtain that: $\frac{\partial}{\partial z}\boldsymbol{S} = \boldsymbol{\Omega}\times\boldsymbol{S}$. In the following, we will discuss the additional phase induced from the rotating boundary conditions.

Based on the approximation of geometric acoustics, the center of the waveguide modes is located off-center about $R_0 = \sqrt{x_0^2 + y_0^2}$ as shown in Supplementary Figure 3(a), where $x_0 = \frac{\int_{\mathcal{S}} x|p|^2 d\boldsymbol{r}^2}{\int_{\mathcal{S}} |p|^2 d\boldsymbol{r}^2} = -0.014$m, $y_0 = \frac{\int_{\mathcal{S}} y|p|^2 d\boldsymbol{r}^2}{\int_{\mathcal{S}} |p|^2 d\boldsymbol{r}^2} = 0$ and $\mathcal{S}$ denotes the cross section of metasurface waveguide. After we rotate the metasurface waveguide namely $\Omega \neq 0$, the effective propagation length will increase in Supplementary Figure 3(b). The effective propagation length will be $\sqrt{L^2 + (\theta R_0)^2}$ in Supplementary Figure 3(c), where $\theta = \Omega L$. Thus, the phase Θ accumulated during rotating metasurface waveguide when $\Omega \neq 0$ will be $\Theta = k\sqrt{L^2 + (\theta R_0)^2} \approx kL + \frac{kR_0^2}{2}\Omega^2 L$. In addition to the unperturbed phase $\Theta_0 = kL$, the addition phase will be $\gamma = \frac{kR_0^2}{2}\Omega^2 L = \frac{kR_0^2}{2L}\theta^2$.

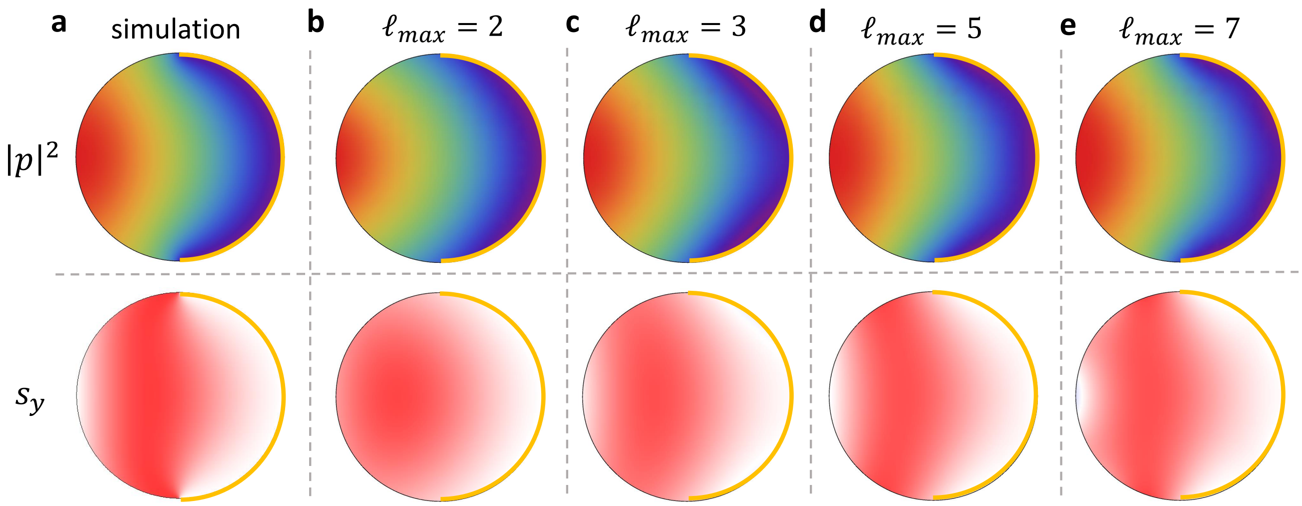

Supplementary Figure 1. **a** The amplitude of the pressured field $|p|^2$ and the spin angular momentum density s_y of the metasurface waveguide eigenmode obtained by numerical simulations. The metasurface boundary is denoted as the yellow color. The $|p|^2$ and s_y obtained theoretically with different $\ell_{\max}$ have been shown in (b-e): (**b**) $\ell_{\max} = 2$; (**c**) $\ell_{\max} = 3$; (**d**) $\ell_{\max} = 5$; (**e**) $\ell_{\max} = 7$.

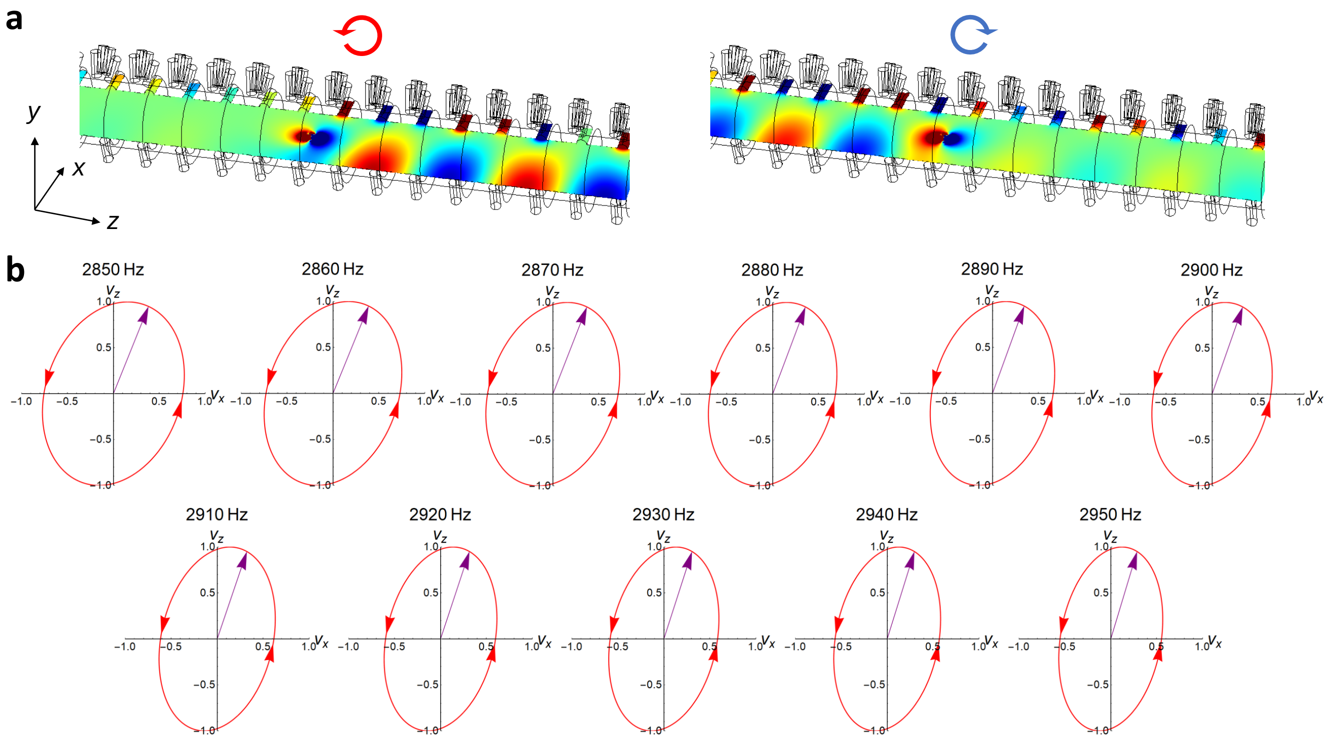

Supplementary Figure 2. **a**, simulations of the selective excitation of metasurface waveguide modes realized by exploiting the spin sources (circularly polarized acoustic dipoles). The excitation frequency is $f = 2.9$kHz. **b**, the simulated velocity field profile at the center of waveguide for $k_z > 0$. The plotted velocity field is normalized by the amplitude of v_z. The purple arrow and the arrowed red circle denotes the polarization of the velocity field and its time evolution. For $k_z < 0$, they will be clockwise elliptically polarized forms due to time-reversal symmetry.

Supplementary Note 4. ROBUSTNESS OF TRANSPORT PROTECTED BY THE SAM

This section will show some cases to demonstrate robust wave transportations protected by the transverse SAM, shown in Supplementary Figure 4. Two cases we have demonstrated: The rectangle metasurface waveguide and circular metasurface

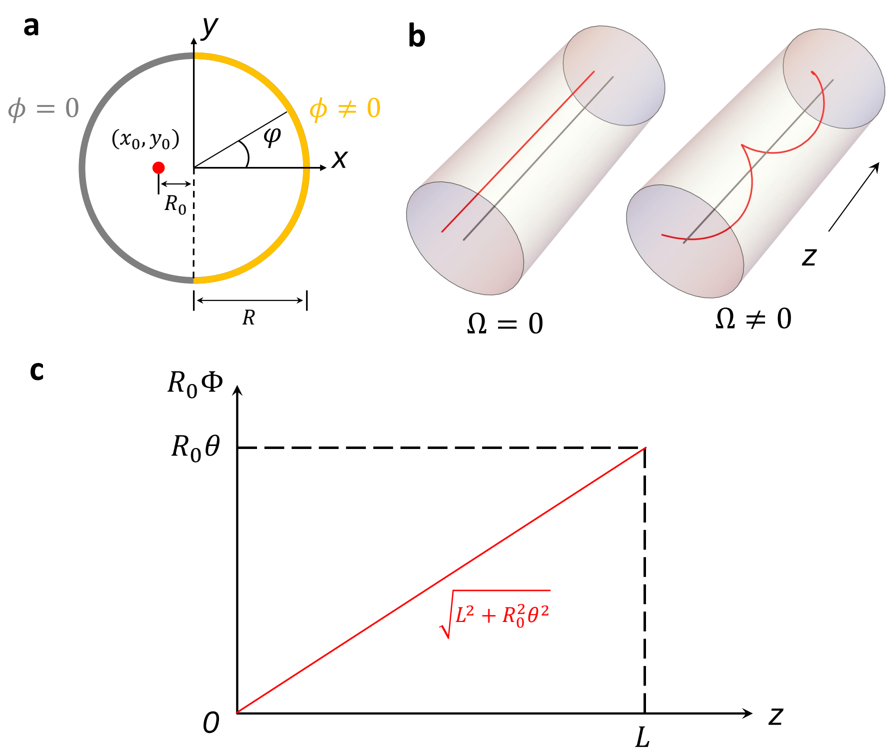

Supplementary Figure 3. **a**, the center position (Red point) of the metasurface waveguide mode with a distance R_0 off-center. **b**, the effective propagation path (Red line) will increase after rotating boundary conditions, namely $\Omega \neq 0$. Gray line denotes the center of the waveguide. **c**, the effective propagation path can be represented by the length of the waveguide L and the arc length $R_0\theta(\theta = \Omega L)$, namely $\sqrt{L^2 + R_0^2\theta^2}$.

waveguide. The reflection phase of the metasurface (denoted as yellow color) is $\phi = \pi$.

In Supplementary Figure 4(a), the width of the rectangle metasurface waveguide is 8cm. The gray and yellow color denote the sound hard boundary (SHB) and the metasurface boundary (MB), respectively. We exploit a strange shape to demonstrate the suppression of corner-scatterings. The demonstration frequency is $f = 2$kHz. For the numerical simulations in Supplementary Figure 4(b), we compared wave transports in metasurface waveguide and conventional waveguide (composed of all sound hard boundaries). We can see that wave scatterings in the metasurface waveguide are efficiently suppressed while the wave in the conventional waveguides will suffer from strong corner-scatterings.

In Supplementary Figure 4(c), the radius of the circular metasurface waveguide is 4cm. From the numerical simulation results in Supplementary Figure 4(d), we can see that compared with strong scatterings in the conventional waveguide, the mode in the metasurface waveguide (with soft boundary) will pass through this devious path successfully with only low scattering loss.

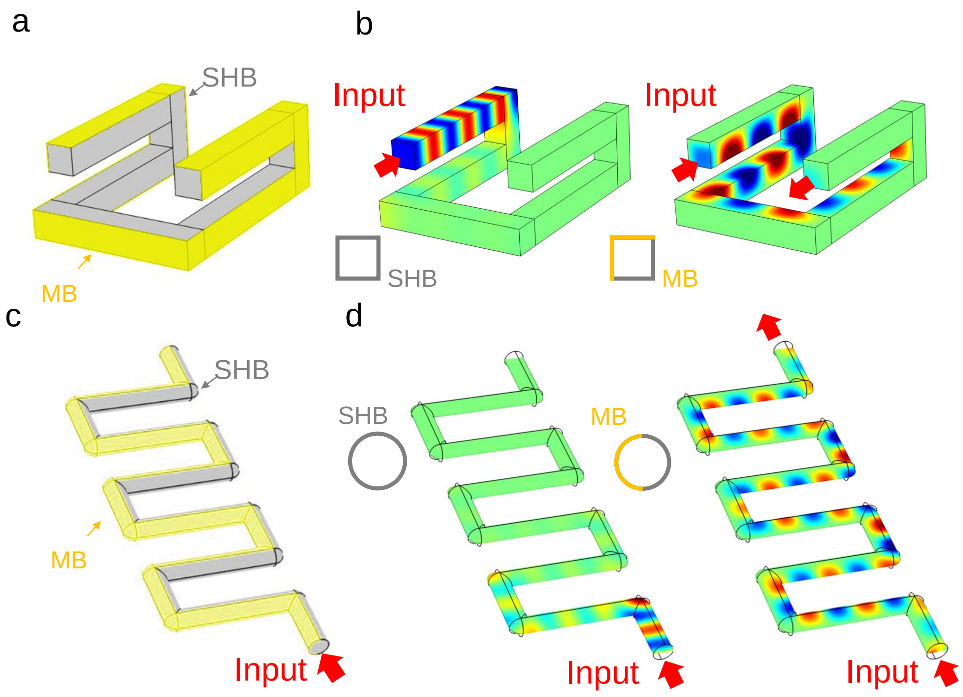

Supplementary Figure 4. The simulation verifications about the robustness of wave transportation if there does not exist the scatters that can flip the SAM texture. The gray and yellow colors in (**a,c**) represent the sound hard boundaries (SHB, the reflection phase $\phi = 0$) and metasurface boundaries (MB, the reflection phase $\phi = \pi$) respectively. The demonstration frequency is $f = 2$ kHz.
